# Functional Anatomy of Ocular Counter-Rolling in Superior Oblique Palsy

**DOI:** 10.1167/iovs.66.12.6

**Published:** 2025-09-03

**Authors:** Joseph L. Demer, Robert A. Clark

**Affiliations:** 1 Department of Ophthalmology, University of California, Los Angeles, Los Angeles, California, United States; 2 Stein Eye Institute, University of California, Los Angeles, Los Angeles, California, United States; 3 Bioengineering Department, University of California, Los Angeles, Los Angeles, California, United States; 4 Neuroscience Interdepartmental Program, University of California, Los Angeles, Los Angeles, California, United States; 5 Department of Neurology, University of California, Los Angeles, Los Angeles, California, United States

**Keywords:** head tilt test, magnetic resonance imaging, strabismus, superior oblique palsy

## Abstract

**Purpose:**

Simulations suggest that displacement of rectus extraocular muscle pulleys in superior oblique (SO) palsy accounts for incomitant strabismus patterns even without postulating SO contractile weakness. We asked how rectus extraocular muscle pulleys reorient during head tilt in SO palsy.

**Methods:**

In 13 subjects with unilateral SO palsy, supine magnetic resonance imaging (MRI) in 2-mm-thick quasi-coronal planes in target-controlled central gaze was repeated in both lateral decubitus positions equivalent to 90° head tilts. From extraocular muscle centroids, we computed oculocentric pulley coordinates and compartmental posterior partial volumes (PPVs) of the rectus and SO muscles.

**Results:**

Validating atrophy, PPV of the palsied SO was smaller than its fellow (*P* < 10^−4^). In fellow orbits, the array of all four rectus pulleys exhibited counter-rotation during head tilt (*P* < 0.03), averaging 5.9°. The palsied pulley array was in both tilts excyclorotated relative to the fellow orbit, particularly by 4° to 5° for horizontal rectus pulleys (*P* < 0.03), and also counter-rotated with head tilt similarly to the fellow orbit. Differential compartmental changes in PPV were significant in the lateral and superior rectus and SO muscles of the fellow orbit that were consistent with observed torsion, but were absent in the palsied orbit.

**Conclusions:**

Similar counter-rotation of the rectus pulley array during head tilt occurs in both eyes in unilateral SO palsy, but superimposed on excyclorotation of the array in the palsied orbit. Differential compartmental change in PPV occurs during head tilt in the lateral and superior rectus muscles of the fellow but not palsied orbit and could augment ocular counter-rolling.

Magnetic resonance imaging (MRI) permits quantitative evaluation of the size and function of the superior oblique (SO) muscle. The normal SO exhibits bilaterally symmetrical bulk that is maximal in mid-orbit, and increases with contraction from supraduction to infraduction,^1^^–^^3^ allowing change in SO posterior partial volume (PPV) to serve as a contractility metric for its action, as is the case for rectus extraocular muscles.^4^ Denervation of other extraocular muscles (EOMs), such as the lateral rectus (LR)^5^ or those innervated by the oculomotor nerve,^6^ rapidly results in EOM atrophy. Because neurectomy of the trochlear nerve in monkeys rapidly induces SO atrophy,^7^ demonstration of atrophy constitutes objective validation of the diagnosis of SO pathology, independent of clinical history and ocular motility findings.

It has become clinically axiomatic that SO palsy^8^^–^^13^ is the most common cause of vertical diplopia or cyclovertical EOM paralysis,^14^ perhaps because SO palsy has been the default diagnosis as established by the clinical three-step test (3ST).^13^ A positive 3ST in unilateral SO palsy consists of ipsilesional hypertropia (HT) in central gaze that is greater in contralesional than ipsilesional lateral gaze and greater in the head tilt ipsilesional than contralesional side.^10^^,^^15^^,^^16^ The presumed basis of the 3ST is that unopposed inferior oblique (IO) muscle activity increases HT in contralateral gaze,^17^ but this surmised mechanism is dubious because MRI demonstrates no greater IO size or contractility.^2^ The increase in HT associated with ipsilesional head tilt is conventionally speculated to result from deficit of the palsied SO incycloduction during ocular counter-rolling (OCR)^16^ that is replaced by ipsilateral superior rectus (SR) muscle contraction,^18^^,^^19^ but there is no direct evidence supporting this mechanism. Nevertheless, when the 3ST is fulfilled, clinicians often reflexively infer SO weakness and attribute variable incomitance of HT to secondary changes.^8^^,^^20^

Surgical treatment of head tilt–dependent HT is mostly beneficial, but no consensus has emerged for optimal strategies,^11^ perhaps in part because fundamental problems confound conventional diagnosis of SOP. Computational modeling has repeatedly confirmed that SO weakness alone cannot account for the large HT common in SO palsy, implicating additional mechanisms.^21^^,^^22^ While aplasia of the trochlear nerve is strongly correlated with hypoplasia of the corresponding SO muscle,^23^ careful imaging in many cases that were clinically diagnosed as congenital SO palsy failed to demonstrate either MRI finding.^24^ Vigorous SO contractile force was directly measured during surgery under topical anesthesia in a case exhibiting a positive 3ST.^25^ The magnitude of the final step in the 3ST, which is the difference in HT between ipsi- and contralateral head tilt, is unrelated to SO size and presumably to SO function.^18^ The most commonly performed strabismus surgery for SOP is IO weakening, which decreases the effect of head tilting on HT, despite the logic of the 3ST predicting the opposite.^26^ Although the 3ST remains widely practiced,^14^^,^^27^ its errors^26^ have been recognized in several common clinical situations, and it is reported that the 3ST is only 70% ^28^ to 75% sensitive^19^ and 50% specific for actual SO atrophy or hypoplasia.^29^ In contrast to this limited reliability, ipsilateral SO volume reduction to no more than 75% of contralateral SO is 99% sensitive and 95% specific for absence of the subarachnoid trochlear nerve.^23^ We introduced the term “masquerading SO palsy” (mSOP) when we described eight congenital and 18 acquired cases fulfilling the 3ST and every other hallmark of SOP, yet exhibiting convincing MRI evidence of normal SO structure and function.^30^ We also showed that abnormal rectus pulley positions^31^^,^^32^ (for example, as in SES^33^) can produce horizontally and vertically incomitant HT consistent with SO palsy. However, in mSOP, pulley locations are bilaterally symmetrical, excluding pulley heterotopy as the cause of HT.^30^

A key element of the 3ST is the effect of head tilt on HT, a stimulus that normally induces OCR as a static vestibulo-ocular reflex. Arising from the otoliths in the inner ear,^34^ OCR is implemented via the neural integrator.^35^ Static OCR is associated with the addition of a constant torsion to all eye positions, shifting Listing's plane along the torsional direction.^36^^–^^40^ Saccades during static OCR are initiated from the shifted Listing's plane.^41^^,^^42^ An ocular torsion of 3° to 7°^43^^–^^45^ results from 90° head tilt relative to gravity, illustrating that the typical gain (eye roll/head roll) of static OCR is only about 0.1 to 0.2 in monkey^37^ and ranges from 0.08^43^ to about 0.10 to 0.27^46^^–^^48^ in humans. Although static OCR maintains torsional eye position of only a few percent of head tilt, dynamic torsional OCR has a much higher velocity gain,^49^ even approaching 100% of head velocity under visual enhancement of the VOR.^50^ However, unlike dynamic tilt, static head tilt can be maintained indefinitely in the lateral decubitus position in an MRI scanner, where it has been demonstrated that static decubitus tilt rotates the EOM pulley array in the direction of OCR by an amount about half the ocular torsional angle.^51^ At the same time, the SO and IO exhibit reciprocal contractile changes appropriate to the ocular torsion.^51^^,^^52^

The effects of head tilt on EOM contractility and pulley positions have been investigated in normal subjects, but there has to date been no MRI investigation of possible abnormalities in cyclovertical strabismus that varies with head tilt. Because the SO orbital layer inserts via the SO sheath on the rectus pulley system, it would seem likely that OCR would be abnormal in SOP that is distinguished by SO atrophy. The current study investigated OCR of the rectus pulleys in SO palsy confirmed by SO muscle atrophy, with the expectation that OCR would be abnormal if dependent on ipsilateral SO muscle function. It was anticipated that OCR might be abnormal in the fellow orbit if central neural adaptation developed in response to contralateral SO weakness.

## Methods

Subjects were 13 volunteers with unilateral SO palsy recruited from the clinical practice of the first author. Subjects gave written informed consent according to a protocol conforming to the tenets of the Declaration of Helsinki and approved by the Human Subject Protection Committee at the University of California, Los Angeles. None of the subjects had history or symptoms of any other neurological disease. None had experienced significant orbital trauma, previously undergone ocular surgery, or had evidence of neurological disease or thyroid ophthalmopathy. Subjects underwent examination of visual acuity, ocular motility, stereoacuity, and ocular anatomy. Binocular alignment was measured by prism-cover testing with appropriate refractive correction for a distant, accommodative target in diagnostic central and secondary gazes, as well as in head tilt to the right and left shoulder. Stereopsis was measured using the Titmus test and subjective cyclotropia using double Maddox rods.

High-resolution, T1 or T2 fast spin echo (T2FSE) MRI was performed using a SIGNA 1.5T MRI scanner (GE HealthCare, Chicago, IL, USA) as described in detail elsewhere,^51^ including the use of a dual-phased surface coil array (Medical Advances, Milwaukee, WI, USA) and central fixation targets. The technique for T2FSE imaging has been published.^53^ All subjects were scanned in 90° right-side-down and left-side-down decubitus postures (Fig. 1), and seven were also scanned in asupine position to confirm that there was subnormal contractile thickening of the ipsilesional SO from supraduction to infraduction; the remaining cases of SO atrophy were unequivocal in central gaze, allowing omission of such functional confirmation in the interest of economy of scanning time. All subjects met the published criterion distinguishing true SOP from mSOP; that is, the maximal ipsilateral SO cross-section in quasi-coronal planes was less than 80% of its fellow, averaging both decubitus head tilts where supine MRI was not performed.^30^ During imaging, the scanned eye monocularly fixated a fine optical fiber illuminated from its distal end by a light-emitting diode in straight-ahead gaze about 2 cm distant. There was no stimulus to convergence. Although each eye was scanned as closely as possible to central gaze position to avoid variations due to vertical eye position, some variation was unavoidable due to the complex surface coil and target positioning in decubitus positions. Actual vertical eye position was therefore measured to verify that it did not confound the main results.

**Figure 1.**
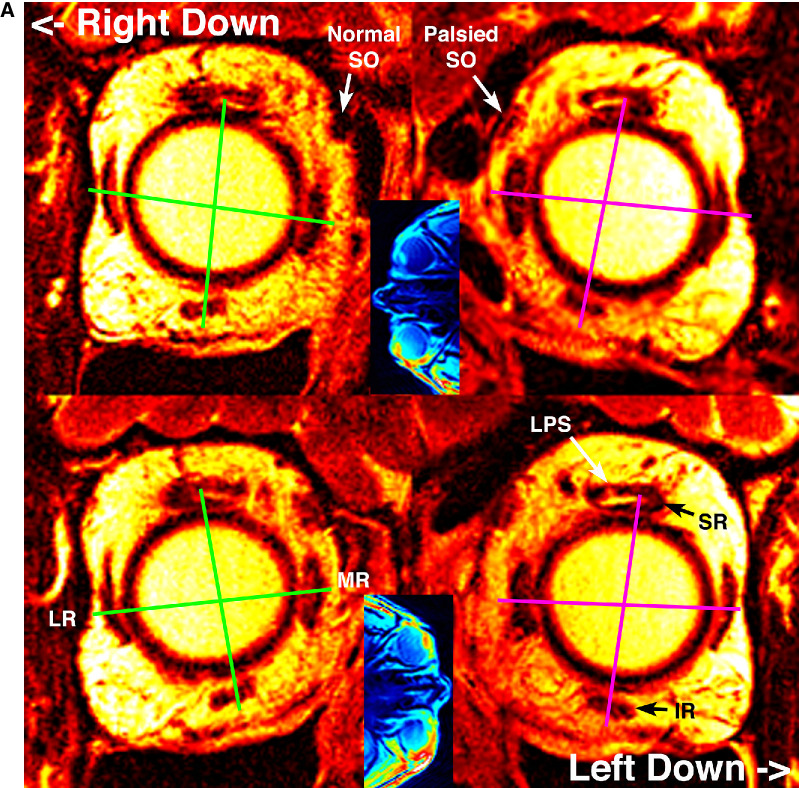
(A) MRIs of a 60-year-old woman with left SO palsy with 8Δ left HT in upright central gaze. Quasi-coronal MRIs near the level of the rectus pulleys in right decubitus (*top row*) and left decubitus (*bottom row*) postures, as indicated by the *blue* veridical axial inset images in the center column. The *green lines* interconnect the corresponding rectus pulley pairs, showing little change in torsional orientation due to head tilt in the left orbit but larger change in the fellow right orbit. Because the anterior SO is tendonous, it does not exhibit atrophy. (B) Quasi-coronal MRIs in mid-orbit demonstrated a reduction in SO cross-section in central gaze and a greater increase in normal right SO cross-section in down gaze than for the left SO. LPS, levator palpebrae superioris muscle; ON, optic nerve.

**Figure 1.**
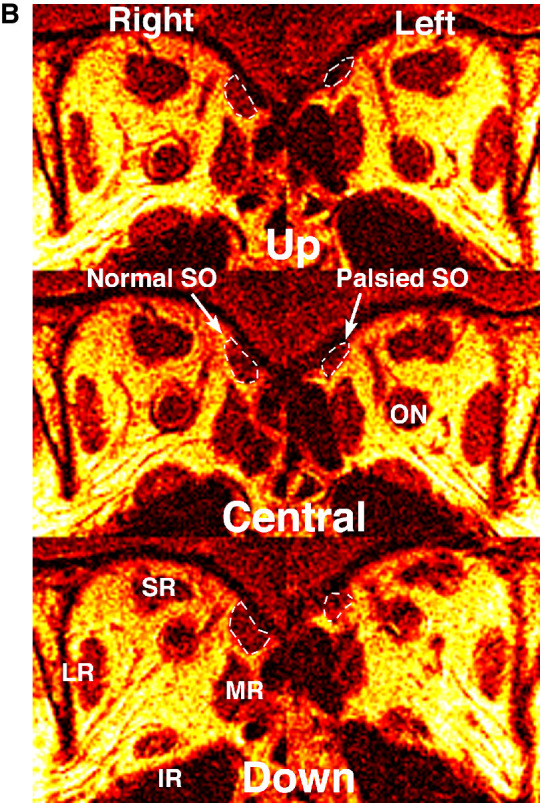
Continued.

A triplanar scan was obtained to verify head positioning in each decubitus position, with correction and repetition as necessary. Sets of 18 to 20 contiguous, 2-mm-thick quasi-coronal images were oriented perpendicular to the long axis of each orbit using a 256 × 256 matrix over an 8-cm field of view, yielding 312-µm resolution. The right decubitus posture was scanned before the left, and both before supine imaging. The right orbit was scanned before the left orbit.

Images in TIFF format were analyzed using the program ImageJ (64-bit) (National Institutes of Health, Bethesda, MD, USA). Images of the left orbit were digitally reflected to the configuration of a right orbit. In quasi-coronal images, rectus EOMs and the SO were manually outlined.^54^^,^^55^ The cross-sectional area of each EOM was automatically determined. The location of each rectus EOM was described by the “area centroid” function of ImageJ. The center of the best sphere fitting the globe was determined using cross-sectional images of the globe in three separate planes.^56^ Rectus EOM paths were then translated to a Cartesian coordinate system originating at globe center taken as positive in the lateral, superior, and anterior directions and rotated into a standard orientation referenced to orientation of the interhemispheric fissure of the brain and the junction of the superior ethmoid air sinus and the orbit.^56^ Data were then scaled to normalize each globe to 24.3-mm average diameter found by MRI in normal subjects,^56^ permitting averaging of pulley positions among subjects^56^ and direct comparison with other studies we have published using identical methods.

The PPV of EOMs was computed from the four image planes beginning 14 mm posteriorly to the globe–optic nerve junction and continuing anteriorly, as this measure has been demonstrated to be the best measure of contractility of multiple alternatives evaluated.^4^ Torsion of pulleys was defined as rotation about the long axis of the orbit, analogous to the definition of ocular torsion as globe rotation around the line of sight. Intorsion constituted medial shift of the SR, inferior shift of the medial rectus (MR), lateral shift of the inferior rectus (IR), and superior shift of the LR.

For rectus EOMs, the angle of a best-fit line through the maximum transverse dimension of its cross-section was computed^52^ and the image rotated to align it to vertical for horizontal EOMs and horizontal for vertical EOMs. Superior and inferior horizontal rectus compartmental PPVs were calculated from cross-sectional areas above and below the perpendicular bisector of that best-fit line, omitting a band 20% of the length of the best fit line to account for anatomical variation in the compartmental border; corresponding medial and lateral vertical rectus compartmental areas were calculated relative to that bisector,^52^ but again omitting the central 20%. For the SO, a published bootstrap analysis showed that a line oblique to the long axis of the SO cross-section discriminates compartmental function, again omitting the central 20% of the cross-section to account for irregularities in the compartmental boundary.^57^

Parametric statistical analyses were performed, including paired two-tailed tests and two-way analysis of variance (ANOVA). Because this was an exploratory study without precedent for SO palsy, it was not possible to estimate sample size a priori.

## Results

### Subjects

Subjects were nine men and four women with SOP with an average age of 41 ± 17 years (range, 15–76). They reported average onset of diplopia at age 22 ± 21 years (range, birth to 66 years). Four subjects gave a history suggestive of congenital onset. Nine cases were acquired, although etiologies were identifiable in only four of the these. Mean central HT was 15 ± 8Δ, increasing to 25 ± 12Δ in contralateral gaze and decreasing to 8 ± 7Δ in ipsilateral gaze. There was an average 23 ± 14Δ HT in ipsilateral versus 4 ± 5Δ in contralateral tilt. Eight subjects 27 to 40 years of age exhibited stereopsis by the Titmus method, including all subjects with congenital onset. Mean excyclotropia by the double Maddox rod method was 6° ± 4°. Eleven subjects exhibited overelevation in adduction of the hypertropic eye, and, ipsilateral to it, 11 exhibited underdepression in adduction. On a customary clinical scale of −4 to +4, mean underdepression in adduction was −1.7 ± 1.3 (range, 0 to −4), and overelevation in adduction was +2.3 ± 1.5 (range, +0 to +4).

### SO Anatomy

**Figure 2.**
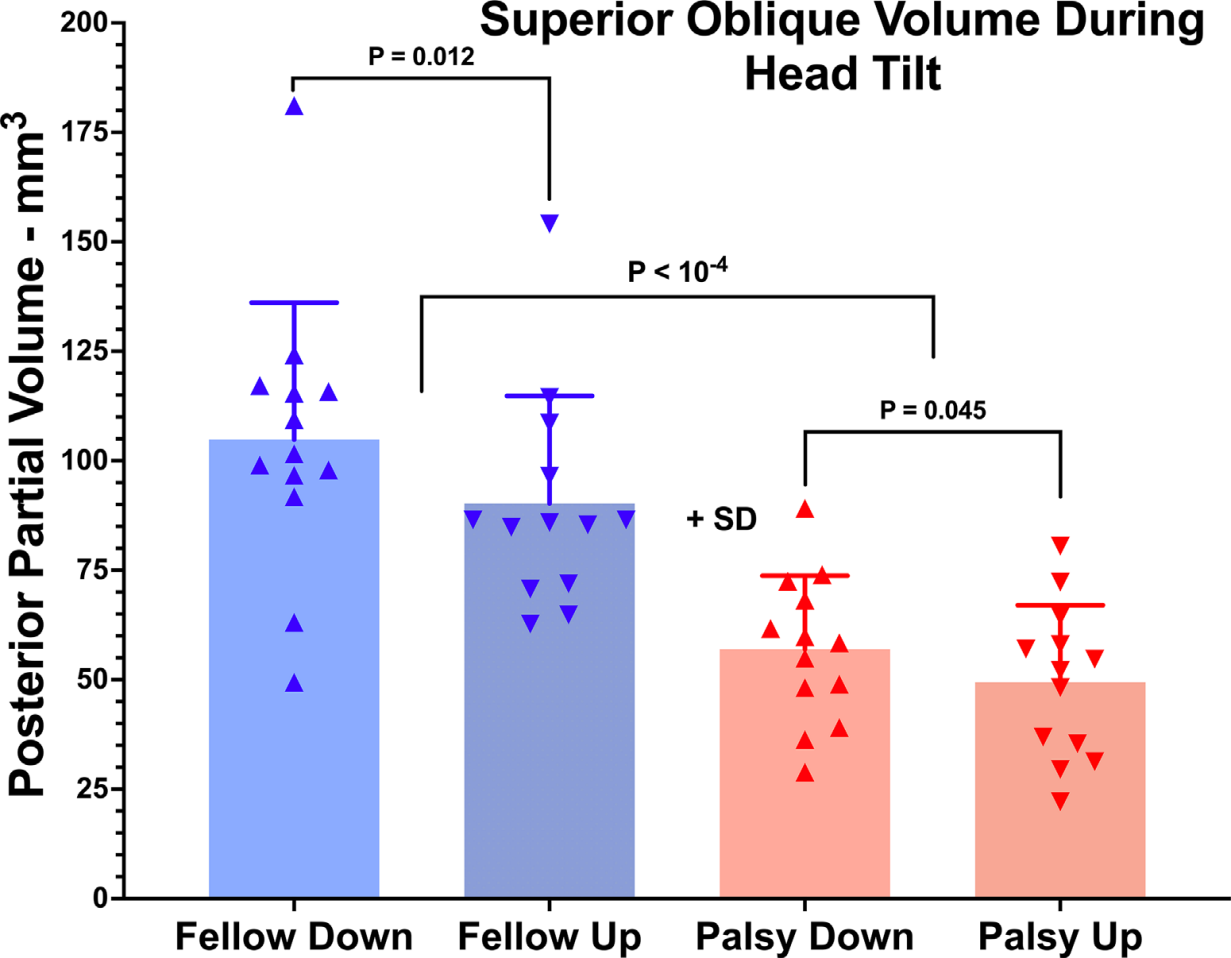
PPV of palsied and fellow SO muscles with their orbits in decubitus postures tilted 90° up and down. Each *symbol* indicates one muscle.

All palsied SO muscle bellies exhibited significantly smaller PPV than their contralateral fellows, a difference that was statistically significant for PPV in both ipsilateral and contralateral tilt positions (*P* < 10^−4^) (Fig. 2). The average ratio of maximum cross-sectional area of the palsied to fellow SO was 0.47 ± 0.16 (range, 0.25–0.74), so that all cases met the published criterion that this value be less than 0.8 in order to exclude mSOP.^30^ In both head tilts, the PPV of the palsied SO averaged 54% of its fellow. These findings validate the demonstration of unilateral SO atrophy confirming the diagnosis of SO palsy, although it was expected based on subject selection. It is notable that SO PPV was greater during ipsilateral tilt for both fellow (*P* < 0.02) and palsied (*P* < 0.05) SO muscles. From upward to downward tilt, PPV of the palsied SO increased 7.5 ± 12.1 mm^3^, whereas there was not a significant change to 14.6 ± 17.9 mm^3^ in the fellow SO (*P* = 0.294, paired *t*-test).

### Rectus Muscle Anatomy

**Figure 3.**
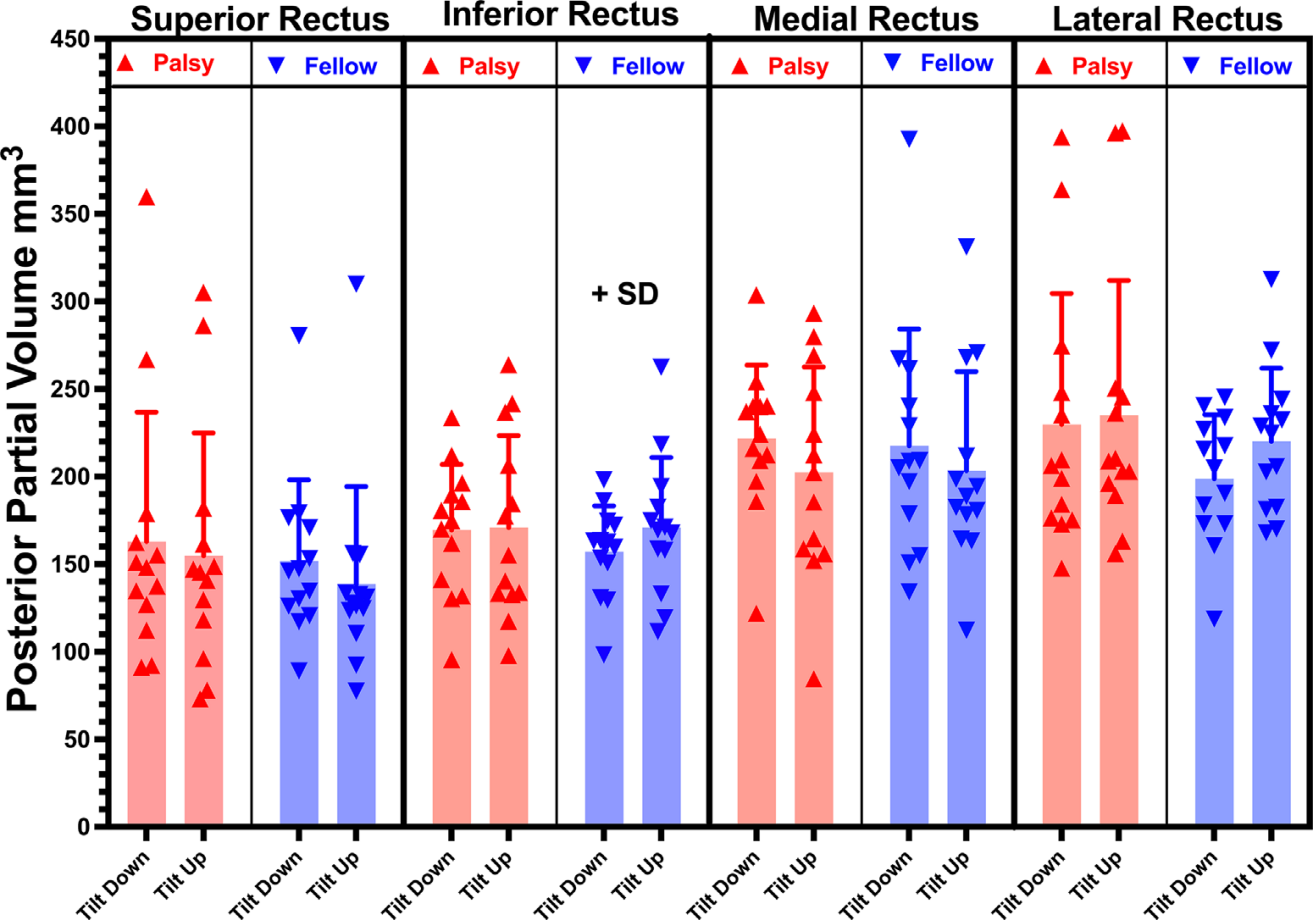
Mean PPVs of whole canonical rectus muscles in orbits of the palsied and fellow SO muscles in decubitus orientation up and down postures. There was no significant variation by ANOVA with head tilt. Each *symbol* represents one muscle.

For each of the four canonical rectus muscles, overall PPV was computed in both decubitus head tilts in both orbits. Neither head tilt orientation nor laterality relative to SO palsy was associated by ANOVA with significant differences in PPV for any whole canonical rectus muscle, as is evident in Figure 3.

### Differential Compartmental EOM Function

**Figure 4.**
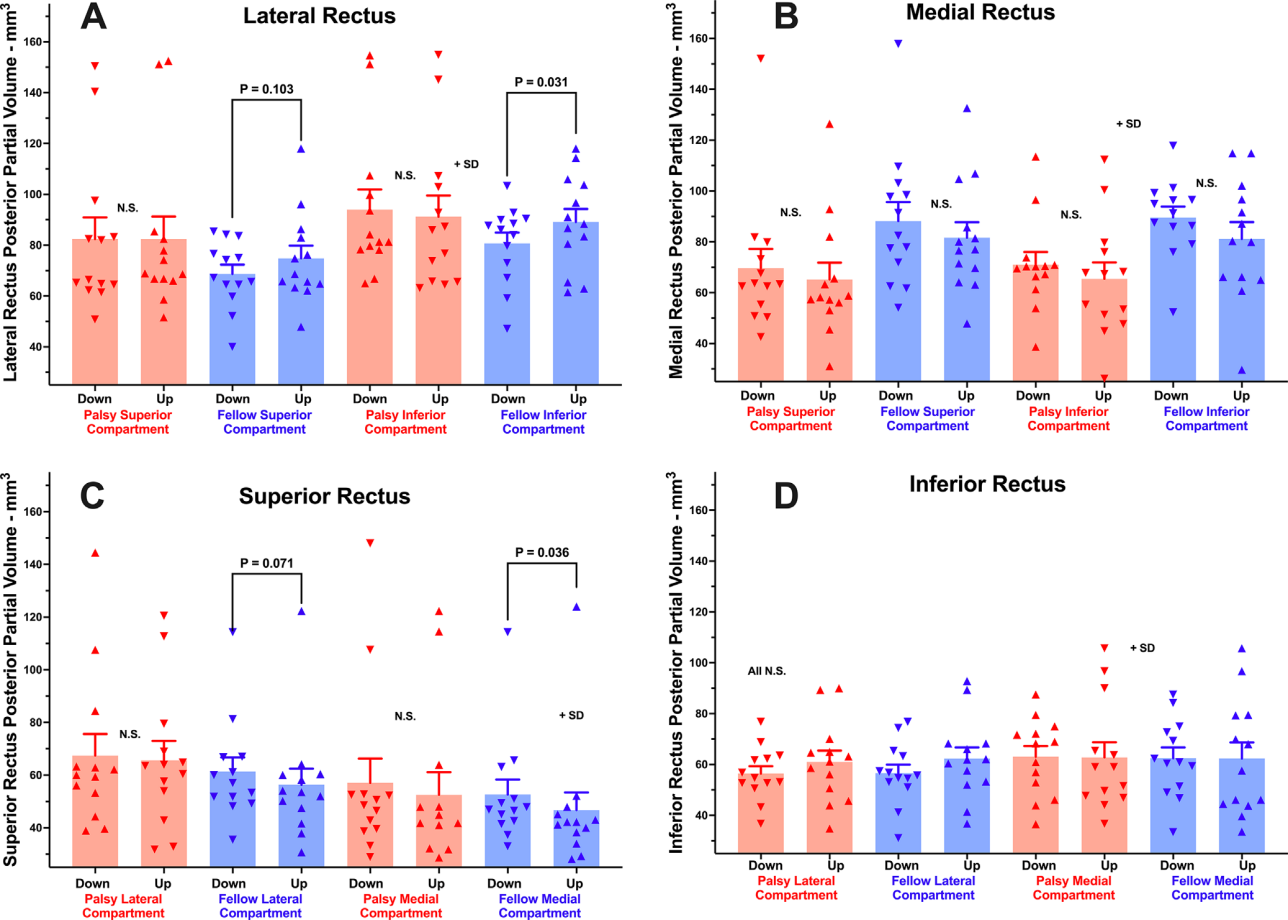
Effect of decubitus head orientation tilting up versus down on mean PPV in the transverse compartments of the lateral (A), medial (B), superior (C), and inferior (D) rectus muscles of palsied and fellow orbits of subjects with SO palsy.

Separate analysis was conducted of PPV changes in the transverse compartments of the four canonical rectus EOMs, excluding the central 20% of the transverse extent of each to avoid misattribution due to variations in compartmental boundaries. Statistical testing was two tailed, except for the special case of the LR, because in normal subjects it has been established that the inferior compartment of the LR contracts in contralateral head tilt to excyclorotate the eye, supplementing the actions of the oblique EOMs.^52^ As illustrated in Figure 4A, PPV of the inferior compartment of the LR was significantly greater when the fellow orbit was tilted upward than downward (*P* = 0.031), but this difference was not significant for the superior compartment (*P* = 0.103). In the palsied orbit, head tilt had no significant effect on PPV in either LR compartment (Fig. 4).

Statistical testing was two tailed for the other canonical rectus EOMs. The medial compartment of the SR of the fellow orbit exhibited greater PPV in downward than upward tilt orientation (*P* = 0.036), but this trend was not significant for the lateral compartment (Fig. 4C). There were no significant differences in PPV associated with head tilt orientation for the transverse compartments of the MR and IR muscles (Figs. 4B, 4D).

**Figure 5.**
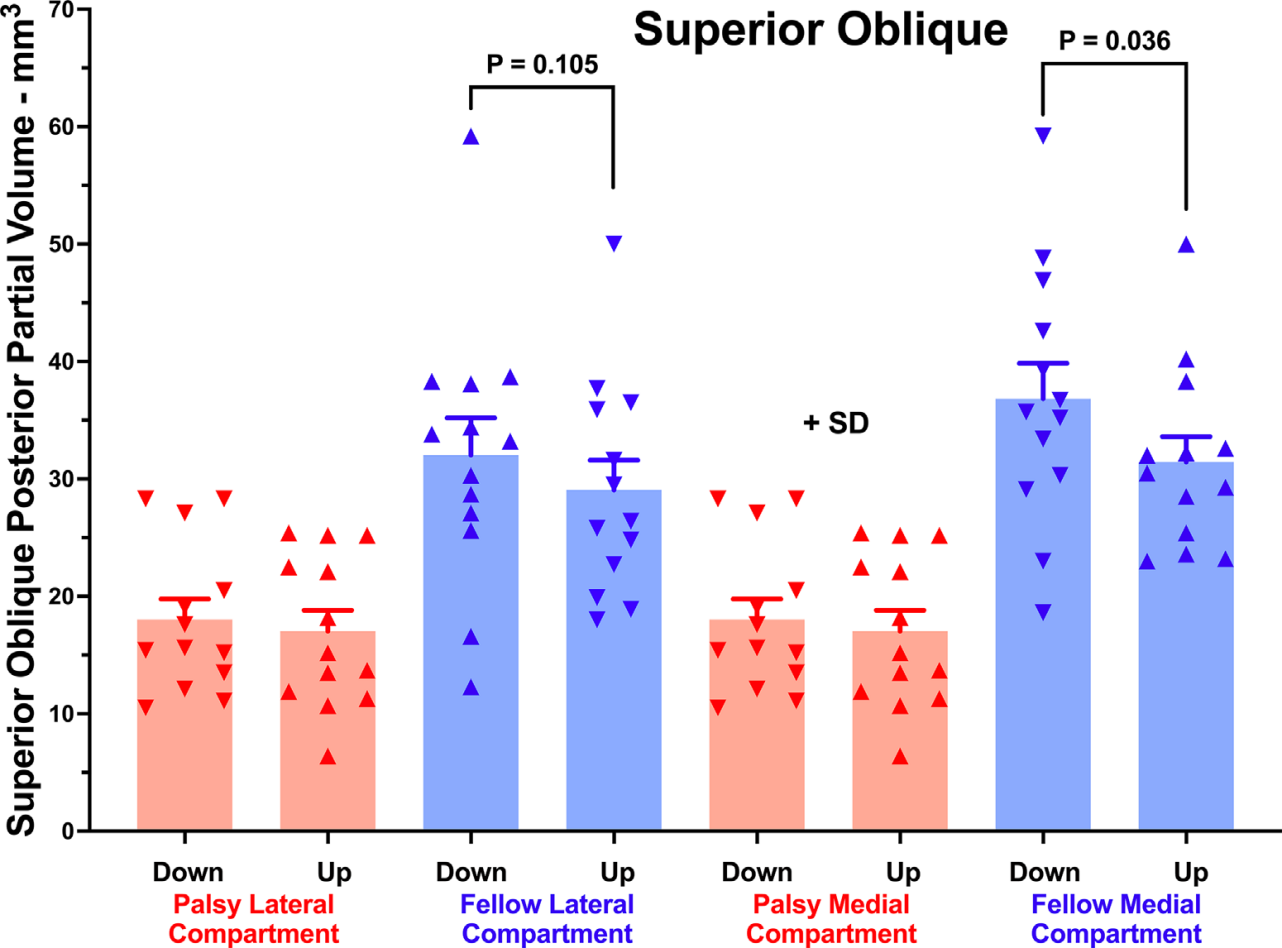
PPVs of the lateral and medial compartments (30° demarcation) of the SO muscle with orbits in downward and upward decubitus orientations, in unilateral SO palsy.

Separate analysis was conducted of PPV changes in the medial and lateral transverse compartments of the SO muscle, with the intercompartmental border taken at 30° to the long axis of the ellipse best fitting the EOM cross-section (Fig. 5). Statistical testing was two tailed in the absence of a prior hypothesis about expected effect. Whereas in the palsied orbit PPV did not significantly differ in the two SO compartments in either head tilt, PPV was significantly greater at 36.8 ± 10.9 mm^3^ with the orbit oriented downward compared with 31.5 ± 7.7 mm^3^ with the orbit oriented upward in the medial compartment of the fellow orbit (*P* = 0.011). In the lateral compartment of the fellow SO, PPV was not significantly different at 32.0 ± 11.5 mm^3^ in downward orientation than 29.1 ± 9.2 mm^3^ in the upward orientation (*P* = 0.105). This comparison also showed no significant difference when analyzed for a 60° angled border between SO compartments (*P* = 0.141).

### Rectus Pulley Positions

**Figure 6.**
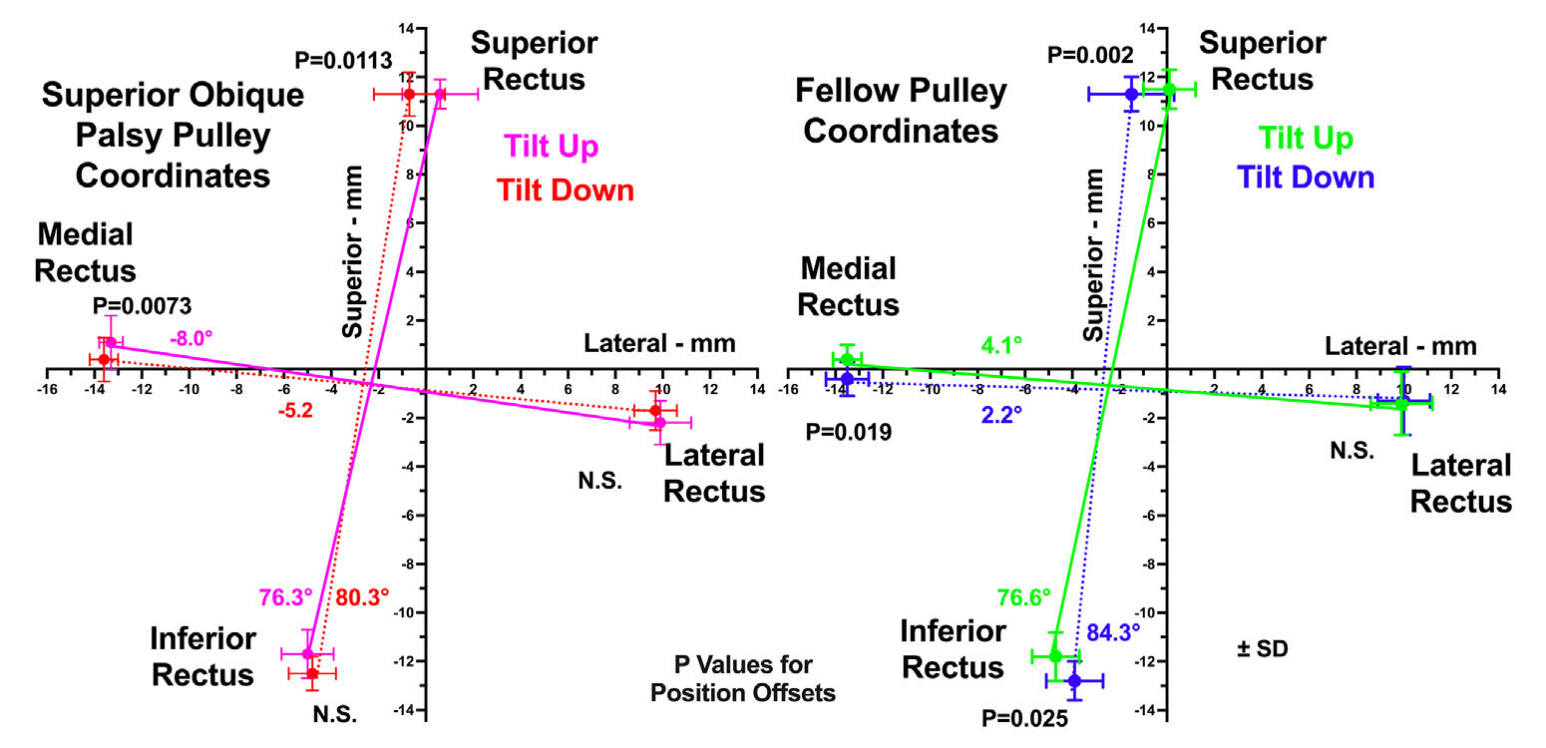
Mean rectus pulley coordinates in palsied and fellow orbits. Note the more excyclorotated orientation of the rectus pulley array in the palsied orbit, yet by paired *t*-test there are significant counter-rotational changes in pulley positions for the MR and SR in both orbits, and the IR in the fellow orbit. The *colored angle values* apply to the lines interconnecting the horizontal and vertical rectus pulley pairs. N.S., not significant.

Locations of the four rectus pulleys are plotted in Figure 6 for the tilt-up and tilt-down postures in both the hypertropic palsied and fellow orbits. The angles denoted in Figure 6 are for line segments connecting the horizontal rectus pairs and vertical rectus pairs, based on differences in the Cartesian coordinates of the respective pairs of pulleys. In both head tilts, it is notable that the rectus pulley array appears more excyclorotated in the palsied than fellow orbit. It is also notable from Figure 6 that the LR pulley position did not change in either orbit with head tilt and that the IR pulley position changed only in the fellow but not the palsied orbit. There is no evidence of LR pulley sag.

**Figure 7.**
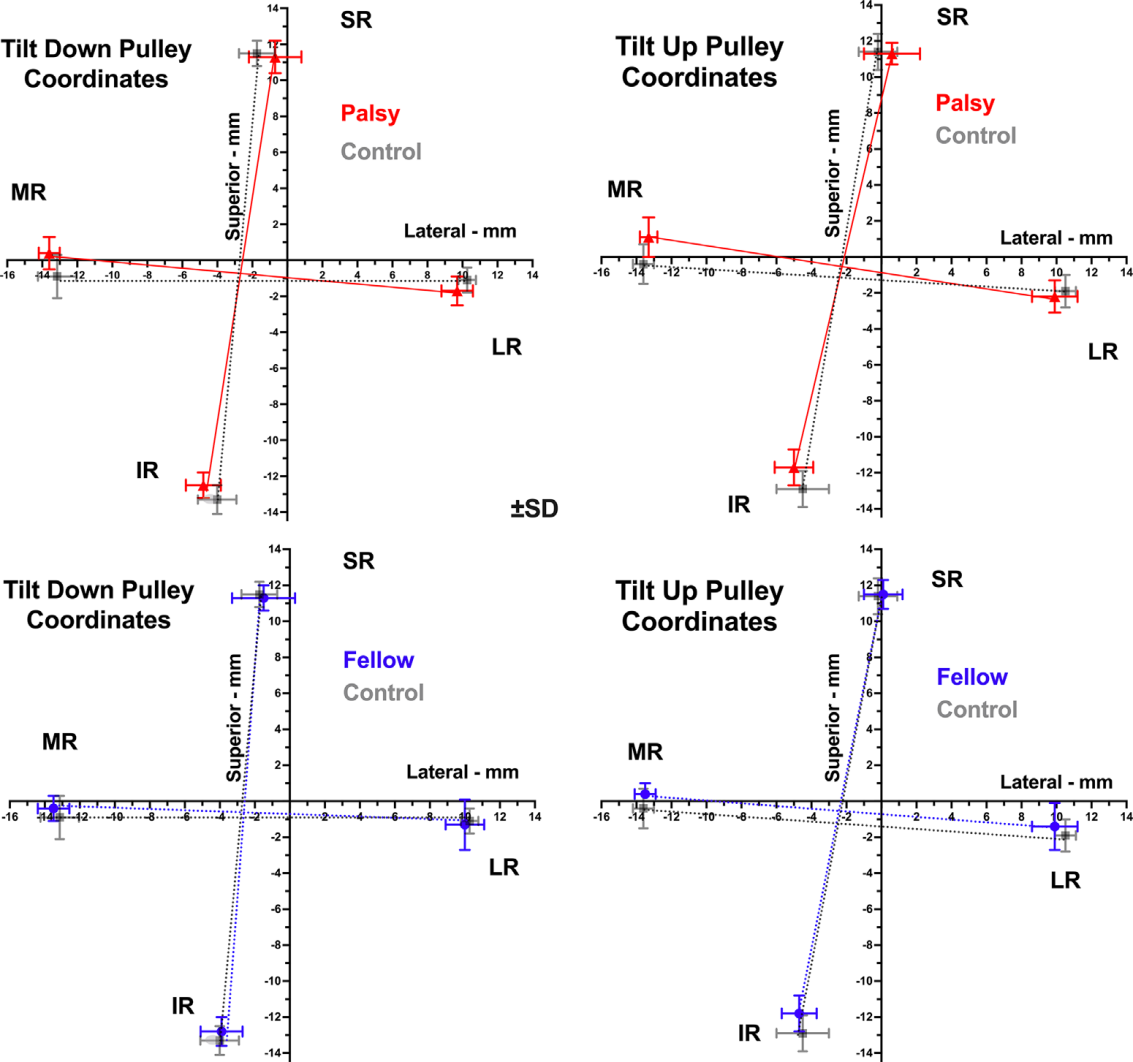
Rectus pulley coordinates in palsied and fellow orbits in tilt-down (*left column*) and tilt-up (*right column*) positions are shown for the palsied orbit in *red*, fellow orbit in *blue*, and published normal controls in *gray*.^51^

It is informative to compare the current data on pulley positions with published data we obtained in normal control subjects using identical technique and analysis. This comparison is illustrated in Figure 7, which superimposes the control data in gray on data for the palsied and fellow orbits in red and blue, respectively. It is evident that, for both head tilts, the pulley coordinates for the fellow orbit of SO palsy generally superimposes on the control data, while the entire pulley array for the palsied orbit is excyclorotated relative to control in both head tilts.

**Figure 8.**
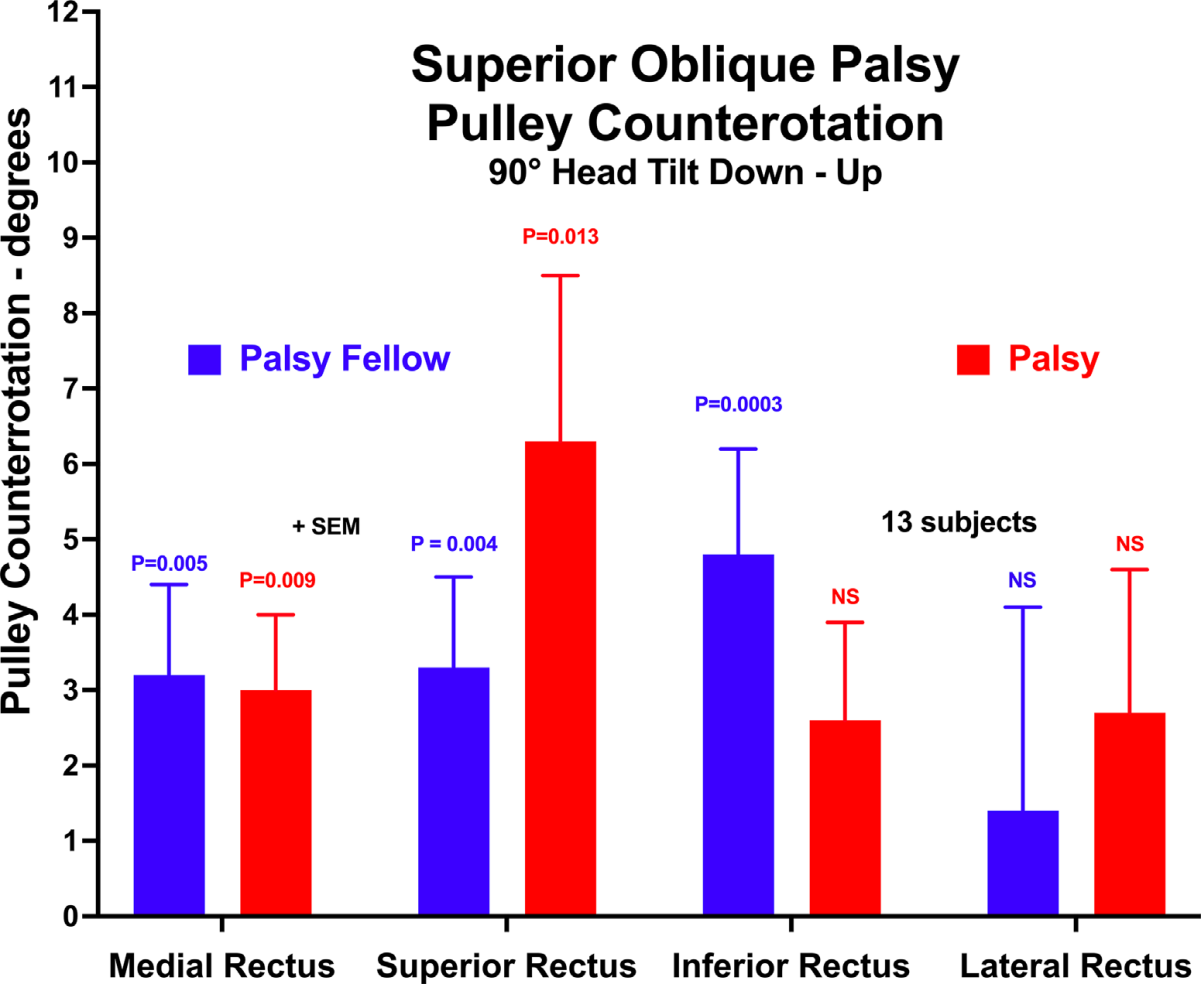
Counterrotation of rectus pulleys due to head tilt in the palsied and fellow orbits. There was significant counter-rotation of the palsied MR and SR pulleys, and the fellow MR, SR, and IR pulleys; *P* values are comparisons with zero. There were no significant effects of canonical pulley or palsy by ANOVA.

A concise way to appreciate changes in the angular orientation of the rectus pulley array is by calculation of their Cartesian angles in the plane of individual pulleys, as illustrated in Figure 8. From tilt down to tilt up, the rectus pulleys of the fellow orbit counter-rotated 1° to 5°, as compared with 2.5° to 6° in the palsied orbit. This counter-rotation was significantly nonzero for all rectus pulleys except the LR in the fellow orbit but was only significant for the MR and SR in the palsied orbit. However, by ANOVA, there was no significant difference in pulley array counterrotation between palsied and fellow orbits (*P* > 0.4).

## Discussion

Subjects in this study who had SOP all exhibited significant HT with strongly positive 3ST changes convincingly fulfilling traditional expectations for unilateral SO palsy, as confirmed by MRI demonstrating SO atrophy. Not only is the 22-year mean age of subjects inconsistent with an alternative cause of positive 3ST, sagging eye syndrome, that typically develops over age 50 years,^58^^,^^59^ but LR sag was also explicitly excluded based on MRI findings to obtain a pure sample of unilateral SO palsy. The current study demonstrated by MRI in lateral decubitus head tilts that OCR of the rectus pulley array occurs similarly in the palsied and fellow orbits in unilateral SO palsy, with magnitude similar to the average value of 4.1° published for healthy normal subjects.^51^ However, in the palsied orbit, the rectus pulley array makes these changes superimposed on an orientation that is significantly excyclorotated, whereas that in the fellow orbit is similar to published normal controls. The current finding thus indicates that the derangement is mainly static, selectively involves only the hypertropic eye, and spares the hypotropic fellow. We have earlier reported that, in SO palsy, the incomitant patterns of variation in HT as tested in upright posture can be obtained by computational simulations that alter only rectus pulley positions, without assuming any SO muscle weakness at all.^32^

What mechanism could drive counter-rotation of the rectus pulleys in SO palsy? One possibility is residual contractility in the palsied SO muscle itself. Changes in PPV are interpretable quantitatively as muscle contractility.^4^ The change in PPV of the SO associated with head tilt was statistically similar in both orbits, although PPV of the palsied SO averaged 54% of that of its fellow in both head tilts. This probably indicates some residual function of the palsied SO. It is also likely that the SO antagonist, the IO muscle, also drives counter-rotation of the rectus pulleys. Although this study did not specifically image IO size or function, the orbital layer of the IO inserts on the inferior and LR pulleys, so that its contraction in infraduction causes excyclorotation of their respective pulleys.^60^ Normally, differential compartmental activation of the LR muscle also contributes to OCR,^52^ and in the fellow but not the palsied orbit the LR superior compartment exhibited greater PPV than the inferior compartment during upward tilt (Fig. 4A). This finding confirms a prior observation suggesting differential compartmental activation contributes to excyclorotation of the rectus pulley array during upward tilt.^52^ Beyond this, the present study observed greater PPV of the medial compartment of the fellow SR during downward tilt (Fig. 4C), which would contribute to incyclorotation of the rectus pulley array and thus work synergistically with differential compartmental behavior in the LR.

The medial compartment of the fellow SO exhibited greater PPV in downward than upward head tilt (Fig. 5), consistent with selectively greater torsional action for the medial compartment whose fibers insert closer to the globe equator than the lateral compartment.^61^ In the lateral compartment of the fellow SO, PPV did not significantly increase in downward tilt. Moreover, there was no differential effect of head tilt on compartmental PPV in the palsied SO muscle. This suggests that the potential for differential torsional–vertical action of SO is lost in SO palsy.

We have elsewhere reported extensive functional studies showing minimal lateral force transmission among arbitrary groups of bovine EOM muscle^62^ and tendon^63^ fibers during external loading, and EOMs actively contracting ex vivo.^64^ We have also published anatomical evidence suggesting only minimal side-to-side junctions among human extraocular muscle fibers.^65^ Patterns of intramuscular innervation appear selective in most canonical EOMs.^61^^,^^66^ In particular, some cases of SO palsy exhibit anisotropic atrophy suggestive of selective compartmental denervation.^67^ These studies provide anatomical and physiological bases for differential compartmental function in human EOMs. Alternative explanations for MRI observations of differential compartmental behavior compartmentalization of EOMs have been offered by some.^68^ The present study, in which torsion was not confounded by change in horizontal or vertical gaze, is not subject to challenge by artifacts of such gaze changes. However, the central neural basis of differential compartmental EOM function remains to be elucidated or the phenomenon excluded.

The current study did not specifically address IO muscle size or function. However, it is elsewhere established by MRI that the IO is neither hypertrophic nor hypercontractile ipsilateral to SOP manifested by significant SO muscle atrophy and that IO size is not correlated with the degree of overelevation in adduction.^2^ Because contraction of the IO is normally associated with OCR of the rectus pulleys,^51^ it is possible that the IO is physiologically the main driver of this phenomenon even when SO function is normal.

A fundamental question remains concerning the 3ST itself. The magnitude of HT in each of the components of the 3ST clearly is not determined by the amount of remaining SO muscle function in SOP, as HT in upright central gaze may vary widely in cases of SO atrophy from 0 to 40Δ.^30^ In particular, the magnitude of change in HT with head tilt is unrelated to size of the SO muscle,^18^ and when the SO muscle is atrophic the HT may range from less than 0 to over 40Δ.^30^ In mSOP where the SO muscle has normal size, HT in upright central gaze may also be up to 20Δ and the magnitude of change in HT with head tilt up to 35Δ.^30^ Both true SOP and mSOP can be associated with HT magnitude reaching the upper limit that can be measured using single ophthalmic prisms. Cases of SES with normal SO size and abnormal rectus pulley locations can be associated with as much as 20Δ HT in upright central gaze and up to 12Δ change in HT with head tilt.^33^ However, both rectus pulley locations and SO muscle function are normal in mSOP, so, although each of these abnormalities may be sufficient for a positive 3ST, neither is necessary. It is therefore proposed that the 3ST does not directly reflect quantitative function of any particular EOM but rather a general disorder of vertical vergence response to otolithic input that may be triggered by known factors such as SO muscle weakness in SOP, imbalance in vertical action of the displaced LR muscle path in SES, or an unknown mechanism in mSOP. This putative general disorder of vertical vergence is proposed to be a central neural mechanism that is distinct from and escapes the usual compensatory mechanisms such as physiologic fusional vergence that ordinarily suppress HT, and may even become markedly enhanced to avert diplopia in intermittent HT.^30^

Listing's law of ocular torsion specifies the relationship between torsional eye orientation and its horizontal and vertical position in the orbit. According to Listing's law, if eye positions are expressed as three-dimensional rotations from primary reference position, the rotation vectors will lie in a plane referred to as Listing's plane. In humans with unilateral SOP, Listing's law continues to be fulfilled, albeit with Listing's plane more temporally rotated in the paretic than in the intact orbit.^69^^–^^71^ In monkeys, acute SOP by subarachnoid trochlear neurectomy caused a large temporal rotation of Listing's plane of the involved eye but did not alter the validity of Listing's law.^72^ This is consistent with continuing action of the rectus pulleys to implement Listing's law, albeit with altered pulley locations as demonstrated here.

Lesion of the neural integrator in the rostral interstitial nucleus of the medial longitudinal fasciculus abolishes the normal torsional shift in Listing's plane associated with OCR.^35^ The interstitial nucleus of Cajal (INC) is the vertical–torsional neural integrator; in monkeys, lesion of the INC completely disrupts OCR.^73^ Moreover, electrical stimulation of the INC in monkeys with pattern strabismus caused by surgery or bolulinum toxin injection in infancy evokes directionally disconjugate eye movements.^74^ However, beyond these observations that indicate that brain structures are involved in OCR and binocular alignment, the specific details of this cyclovertical vergence disorder remain mysterious. The concept of a central vergence disorder is similar to the proposal of Guyton that cyclotorsional strabismus may sometimes be due to a motor fusion disorder and altered EOM lengths.^75^ Interestingly, horizontal saccades are slightly slowed in lateral decubitus positions that evoke OCR.^45^

Development and visual experience may also play roles in the 3ST response in SOP. The current subjects included four congenital and nine acquired cases of SOP, although etiologies were identifiable in only four acquired cases. This lack of specific etiology for SOP is unsurprising, because we have previously reported that etiology and time of onset of head tilt–dependent HT is frequently indeterminate.^29^ Time of symptom onset of mSOP is also often indeterminate and often congenital,^30^ and both true SOP and mSOP respond similarly to strabismus surgery.^76^ Longstanding cases of both true SOP and mSOP may exhibit markedly enhanced vertical fusional vergence range, suggesting that this compensatory phenomenon is due to chronicity and probably gradual progression of HT.^30^ It is also likely that compensatory mechanisms could act covertly to prevent subjects from appreciating the gradual onset of SO weakness or hyperphoria until severe enough to become symptomatic for diplopia. Such considerations do not strongly suggest existence of a critical age period for compensation of HT.

It is informative to note that, in an animal model of complete SO palsy by acute trochlear neurectomy, the initial magnitude of HT was moderate but incomitant, but initially increased and then decreased as it became horizontally concomitant during the initial period when the lesioned eye was continuously patched.^77^ When binocular vision was permitted the HT and its lateral incomitance progressively increased.^77^ In this animal model of SOP, HT changed following the acute lesion, but the cyclotropia did not. This experimental study makes it clear that SO muscle function alone does not determine the degree or comitance of HT in SO palsy, phenomena substantially determined by other mechanisms that evidently do not require abnormal SO function at all. Moreover, counter-rolling of the rectus pulley is to a large extent preserved in both orbits in SO palsy, although in the context of a static extorsional bias in the ispsilesional orbit.
